# The Bright and Dark Sides of Herbal Infusions: Assessment of Antioxidant Capacity and Determination of Tropane Alkaloids

**DOI:** 10.3390/toxins15040245

**Published:** 2023-03-27

**Authors:** Ana Rita Soares Mateus, Carmen Crisafulli, Matilde Vilhena, Sílvia Cruz Barros, Angelina Pena, Ana Sanches Silva

**Affiliations:** 1 National Institute for Agrarian and Veterinary Research (INIAV), I.P., Rua dos Lagidos, Lugar da Madalena, 4485-655 Vila do Conde, Portugal; 2 University of Coimbra, Faculty of Pharmacy, Polo III, Azinhaga de Santa Comba, 3000-548 Coimbra, Portugal; 3 LAQV, REQUIMTE, Laboratory of Bromatology and Pharmacognosy, Faculty of Pharmacy, University of Coimbra, Polo III, Azinhaga de Stª Comba, 3000-548 Coimbra, Portugal; 4 Center for Study in Animal Science (CECA), ICETA, University of Porto, Apartado, 4050-346 Porto, Portugal; 5 Associate Laboratory for Animal and Veterinary Sciences (Al4AnimalS), 1300-477 Lisbon, Portugal

**Keywords:** herbal infusions, tropane alkaloids, validation, QuEChERS, ultra-high performance liquid chromatography, time-of-flight mass spectrometry, antioxidant capacity

## Abstract

Herbal infusions are highly popular beverages consumed daily due to their health benefits and antioxidant properties. However, the presence of plant toxins, such as tropane alkaloids, constitutes a recent health concern for herbal infusions. This work presents an optimized and validated methodology based on the QuEChERS (Quick, Easy, Cheap, Effective, Rugged, and Safe) extraction procedure followed by Ultra-High Performance Liquid Chromatography combined with Time-of-Flight Mass Spectrometry (UHPLC-ToF-MS) for the determination of tropane alkaloids (atropine, scopolamine, anisodamine, and homatropine) in herbal infusions, in accordance with criteria established by Commission Recommendation EU No. 2015/976. One of the seventeen samples was contaminated with atropine, exceeding the current European regulation regarding tropane alkaloids. In addition, this study evaluated the antioxidant capacity of common herbal infusions available on Portuguese markets, indicating the high antioxidant capacity of yerba mate (*Ilex paraguariensis)*, lemon balm (*Melissa officinalis*), and peppermint (*Mentha x piperita*).

## 1. Introduction

Since ancient times, herbal infusions have been popular beverages and have been recognized as an essential source of antioxidants. These compounds, including phenolic acids and flavonoids, are linked to reducing the production of reactive oxygen species (ROS) and oxidative stress, preventing diseases including cancer, cardiovascular diseases, and diabetes [1,2,3,4].

Herbal infusions can be produced using a variety of plants and plant components such as flowers, leaves, bark, and roots. For example, chamomile (*Matricaria chamomilla* L.), lemon balm (*Melissa officinalis* L.), mate (*Ilex paraguariensis* L.), milk thistle (*Silybum marianum* L.), narrow-leaved purple coneflower (*Echinacea angustifolia* DC.), peppermint (*Mentha x piperita* L.), and Greek lemon verbena (*Aloysia citrodora* L.) are popular herbs usually prepared as infusions, due to their organoleptic properties.

In particular, tea is one of the most popular beverages worldwide. Tea is derived from the *Camelia sinensis* L. plant and can be consumed as green tea, oolong tea, or black tea, according to the degree of fermentation [5,6]. Green tea is a rich source of polyphenols, such as catechins, flavones, anthocyanins, and phenolic acids [6,7,8].

Furthermore, since herbal infusions are natural sources of bioactive compounds, they are used by food, pharmaceutical, and cosmetic industries to meet consumers’ demands for more natural products [9,10].

Despite the benefits, there are safety concerns about chemical contaminants in herbal infusions such as pesticide residues, mycotoxins, heavy metals, and plant toxins [11,12,13,14]. Considering plant toxins, tea and herbal infusions are the main contributors to human exposure to pyrrolizidine alkaloids (PAs), due to frequent and high consumption and might represent a risk to human health [15]. The maximum level of PAs is defined for tea and herbal infusions as the lower bound sum of 21 compounds, considering their genotoxic and carcinogenic potential [16].

Tropane alkaloids (TAs) are another kind of emerging plant toxin in tea and herbal infusions. Tropane alkaloids are naturally occurring compounds, produced as secondary metabolites in several species of plants, being the most prevalent in the *Solanaceae* family [17]. In plants, there are more than 200 individual TAs that are generated as a defense mechanism against herbivores, microorganisms, and other plants [18,19].

Atropine and scopolamine are the most researched TAs due to their application in the pharmaceutical industry, since they act as non-selective inhibitors of the muscarinic acetylcholine receptors [20,21]. For instance, atropine is a pre-anesthetic, while scopolamine, in form of butylscopolamine, is used to relieve discomfort associated with spasms of the gastrointestinal tract [20,22]. Atropine presents as a racemic mixture of isomers (−)-hyoscyamine and (+)-hyoscyamine, in which only the (−)-hyoscyamine enantiomer exhibits anticholinergic activity [23].

However, TAs are toxic when ingested in higher doses, decreasing the production of secretions in the digestive and respiratory tract, and causing mydriasis, muscle spasms, and tachycardia [24]. Nonetheless, no chronic or genotoxic toxicity was exhibited [23]. The European Food Safety Authority (EFSA) has defined an acute reference dose (ARfD) of 0.016 g/kg body weight (bw), expressed as the sum of atropine and scopolamine [23], due to the high toxicity of these substances and the frequency of cases of intoxication [25]. Likewise, there are other tropane alkaloids, such as anisodamine and homatropine, which had the same effects but with less toxicity [23].

TAs are predominately found in Atropa belladonna and Datura stramonium and are distributed over all parts of the plant [18]. Due to climate change, those plants grow in new areas as invasive plants, and their leaves, flowers, pericarp, roots, and seeds constitute impurities in crops [17,26]. Therefore, they are emerging natural contaminants, and their occurrence tends to increase. Infusions prepared with dried tea and herbs contaminated with weeds producing TAs contribute to exposure to TAs in humans.

Recently, the European Commission adopted maximum levels for the total of atropine and scopolamine for herbal infusions, applicable from 1 September 2022 [27], in order to achieve the highest standard of food safety because the presence of TA in products is a health concern. Regulation No. 2021/1408, amending Regulation No. 1881/2006 [27], established the maximum level for herbal infusions in dried (25 µg/kg) and liquid (0.20 µg/kg) products. A higher limit of 50 µg/kg was set for dried anise seeds.

Since January 2020, the Rapid Alert System for Food and Feed (RASFF) has reported 49 notifications for bio-contaminants in herbs and spices. Most of the notifications reported the presence of pyrrolizidine alkaloids in aromatic herbs. One notification of high content of tropane alkaloids in peppermint from Turkey was notified in July of 2020 in Germany, reporting a level of 66.7 µg/kg for the sum of atropine and scopolamine [28].

Due to growing evidence that TAs are present in food products and that they may have a negative impact on human health, it is of outmost importance develop methods to determine these chemical contaminants to ensure food safety and protect consumers’ health. In the scientific literature, some analytical methods for the determination of TAs in tea and herbal infusions have been developed in recent years, using different approaches (Table 1).

Regarding the extraction procedure, solid-phase extraction (SPE) is the most widely applied technique for the determination of TAs in dried herbal infusions, as shown in Table 1 [29,34,35]. Moreover, González-Gómez et al. [35] applied a solid phase microextraction (SPME) using μSPEed^®^ cartridges to determine atropine and scopolamine in liquid tea and herbal infusions. The miniaturization of the SPE technique allows the reduction of costs and solvents and a decrease in waste generation, towards a greener extraction procedure [36].

Nevertheless, the QuEChERS (acronym of Quick, Easy, Cheap, Effective, Rugged, and Safe) method initially applied for the determination of pesticide residues, namely in plant-based matrices, and now applied to other food contaminants such as mycotoxins and plant toxins, allows for the rapid analysis of several samples and offers a simple, quick, easy, and environmentally friendly approach to extracting compounds, allowing for effective extraction, with less use of reagents, solvents, and materials, and removing interferents [37]. Despite its outstanding performance in many matrices and for different analytes, the use of QuEChERS for TA determination in herbal infusions is limited [30].

The methods for the detection and/or quantification of TAs in tea and herbal infusions samples include liquid chromatography (LC) coupled with high-resolution mass spectrometry (Table 1), with high selectivity and sensitivity. Most of the studies determine the presence of atropine and scopolamine, or together with pyrrolizidine alkaloids. Moreover, other recently developed methods focus on the determination of TAs in cereals [31,32,38] and leafy vegetables [39].

To fill the gap in multi-analyte methodologies for TAs in this matrix, the main goal of this paper was to develop and validate a QuEChERS followed by Ultra-High Performance Liquid Chromatography combined with Time-of-Flight Mass Spectrometry (UHPLC-ToF-MS) method for detection and quantification of atropine, scopolamine, anisodamine, and homatropine in dried herbal infusions. Furthermore, using the suggested method, this study measured the level of TA in tea and herbal infusions in samples collected in Portuguese supermarkets, improving data on the incidence of these TAs and assessing compliance with EU legal limitations. Another goal of this study was to determine the antioxidant capacity of tea and herbal infusions.

## 2. Results and Discussion

### 2.1. Development and Validation of an Analytical Method for the Determination of TAs

#### 2.1.1. Optimization of Extraction Procedure Conditions

**Figure 1:**
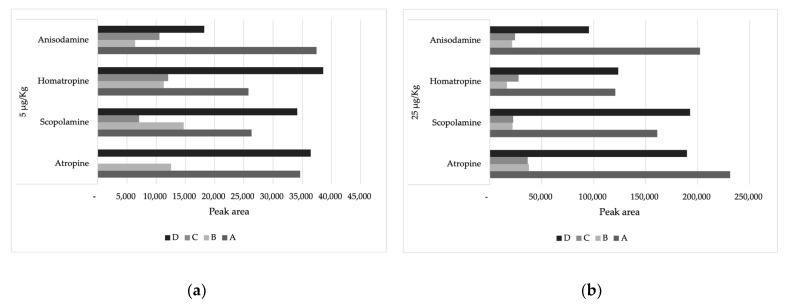
Average peak areas (*n* = 2) using four different extraction procedures for TAs in herbal infusions at the spiked level of (**a**) 2.5 µg/kg and (**b**) 25 µg/kg, where **method A** corresponds to extraction with 10 mL of ACN; **method B** corresponds to use 10 mL of water and 10 mL of ACN; **method C** is the same as B, but with a shaking period of 30 min; and finally, **method D** corresponds to extraction with 10 mL of ACN and shake time (30 min).

The extraction of tropane alkaloids from dried herbal infusions was optimized using a modified QuEChERS (Quick, Easy, Cheap, Effective, Rugged, and Safe) technique. The extraction solvent was optimized for herbal infusions. The influence of different solvents (ACN and water) and time of shaking (0 and 30 min) was assessed by comparing the peak areas for all toxins in a blank sample of herbal infusions spiked at 2.5 and 25 µg/kg (Figure 1). Generally, method D, which used 20 mL of ACN and 30 min of shaking, made it possible to obtain the greatest peak areas and therefore was considered the best method for all analytes. The only exception was for anisodamine, where the method using just ACN, without shaking, had the highest peak area. However, since for herbal infusions there are only maximum levels established for the sum of atropine and scopolamine [26,27], the method was optimized to have the best analytical performance for these tropane alkaloids. The use of water (methods B and C) decreases the extraction efficiency, with a significant reduction of peak areas.

In terms of partition salts, the standard QuEChERS method, based on EN 15662 method for pesticide residues, uses 4 g of MgSO_4_, 1 g of NaCl, 1 g of trisodium citrate, and 0.5 g of disodium citrate, for a 10 g sample. Because the amount of the sample in our approach was lowered to 2.5 g, we also attempted to reduce the amount of salts utilized in the partition. Three different salt quantities were tried for this: 6.5 g, 3.25 g, and 1.62 g, while retaining the original proportions of the four chemicals in the combination. The method using 1.62 g provided better results (data not shown), so it was selected for further analysis. The developed method used four times less of the sample than the classical QuEChERS; for this reason, using four times less of the salts was the best proportion to achieve the best performance. 

The sample preparation included a clean-up step based on d-SPE to remove interferents and include an evaporation step to concentrate the TAs before the chromatographic step. The clean-up step is a very important step to reduce the interferents since herbal infusions are complex matrices due to the presence of chlorophylls.

#### 2.1.2. Validation of Analytical Method

Since there is currently no specific regulation or guidance document for the validation protocol of a method for the quantification of tropane alkaloids or other plant toxins in food products, the optimized method was validated in accordance with the criteria defined by the Decision of 12 August 2002 implementing Council Directive 96/23/EC [40], concerning the performance of analytical methods and the interpretation of the results.

Furthermore, the method was developed taking into consideration the maximum levels of tropane alkaloids in herbal infusions, defined as the sum of atropine and scopolamine. These levels were established by the Commission Regulation (EU) 2021/1408 of 27 August 2021, in effect from 1 September 2022 [27]. The method included atropine (racemic mixture of (−)-hyoscyamine and (+)-hyoscyamine) and scopolamine, as the main tropane alkaloids, considering the EFSA Scientific Opinion on tropane alkaloids [23].

Even though there are no legal limits on anisodamine and homatropine in herbal infusions, it should be highlighted that these two TAs were included in this method, following the recommendations of the EFSA, not only because of the absence of data on their presence in food but also because more than 200 known TAs were found in different plants. To avoid unintentional human exposure to TAs, it is crucial to check the presence of these plant toxins in herbal infusions. Advanced techniques for the detection of tropane alkaloids in tea and herbal infusions should be validated in order to improve the database on the presence of tropane alkaloids in food and ensure food safety.

For correction of signal variations, internal standards (IS) were added at the beginning of the extraction procedure, allowing for the measurement of the relative response ratio between a TA and IS. The isotope-labeled forms of the analyte are more suitable since they will have characteristics similar to the toxin and, consequently, behavior during the analytical method. Along these lines, D5-atropine and 13C,D3—scopolamine were selected as IS for atropine and scopolamine, respectively. Due to the similar structure, D5-atropine was selected as IS for homatropine and 13C,D3—scopolamine for anisodamine.

##### Linearity and Sensitivity 

For the four TAs, the linearity was assessed through the determination coefficients (r^2^) of calibration curves (Table 2) in the constructed matrix-matched calibration curves in different ranges. The determination coefficients were always higher than 0.993, except for anisodamine (r^2^ = 0.9867). The determination coefficient was higher for scopolamine (r^2^ = 0.9976), followed by atropine (r^2^ = 0.9958) and homatropine (r^2^ = 0.9930). All the residuals were always lower than 20%. These results indicate the suitability of the developed method to quantify TAs in the selected calibration range. The method was found linear in the concentration range between 5 and 50 µg/kg for atropine, scopolamine, and homatropine and between 15 to 50 µg/kg for anisodamine.

**Figure 2:**
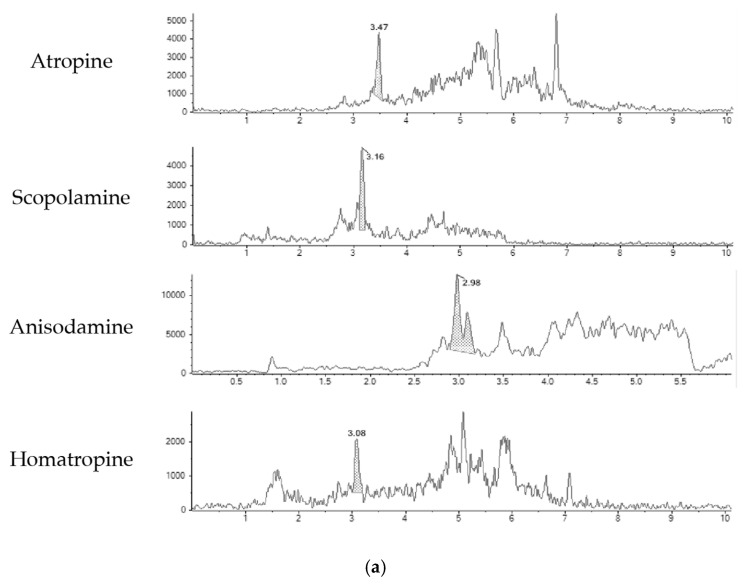

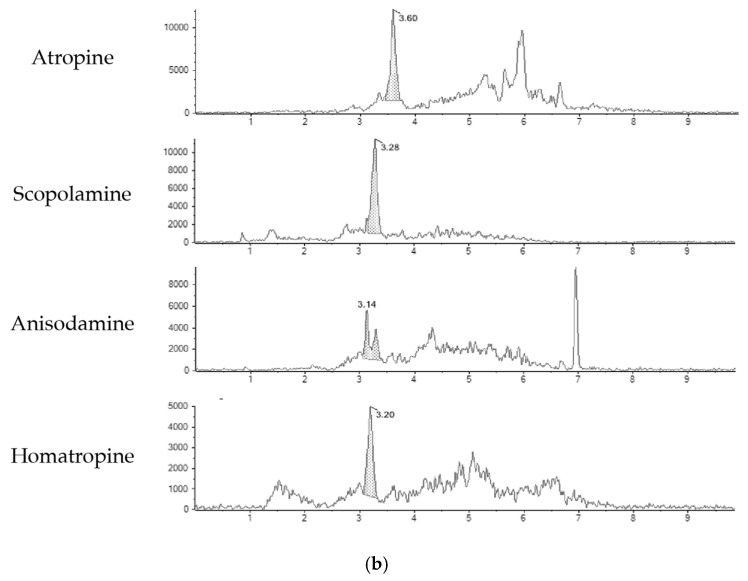
(**a**) Chromatograms of blank green tea sample spiked at LOQ level for each tropane alkaloid, corresponding to 5 μg/kg for atropine, scopolamine, and homatropine and 15 μg/kg for anisodamine. (**b**) Chromatograms of blank green tea sample spiked at 20 μg/kg level for each tropane alkaloid, including atropine, scopolamine, anisodamine, and homatropine.

The outcomes of the method validation show that the LOD and LOQ found (Table 2) indicated that the method is sensitive enough to detect and quantify atropine and scopolamine in dried herbs for infusion, meeting the requirement imposed by EU regulations for the ML of the sum of atropine and scopolamine (25 µg/kg). Additionally, the method is sensitive enough to identify the unregulated anisodamine and homatropine found in herbal infusions. However, the method is equally sensitive for atropine, scopolamine, and homatropine, and less sensitive for anisodamine, which had a higher LOQ (15 µg/kg). The LOQ of 5 µg/kg for atropine and scopolamine is 2.5 lower than the ML, considering half of the ML for each TA. These LOQs are in agreement with the criteria set by the Recommendation (EU) 2015/976 on the monitoring of the presence of tropane alkaloids in food, where the “LOQs should be preferably below 5 μg/kg and not higher 10 μg/kg for agricultural commodities, ingredients, food supplements, and herbal teas and lower than 2 μg/kg for finished foods” [41]. The chromatograms for each tropane alkaloid, at LOQ level and spiked level of 20 μg/kg, are presented in Figure 2.

Compared with other studies, presented in Table 1, a procedure based on QuEChERS developed by León et al. [30] provided a similar LOQ for atropine and scopolamine. The results obtained in this study are comparable to those reported by Romero-Torres et al. [34] (LOQ: 5–15 μg/kg) and González-Gómez et al. [29] (LOQ: 1.9–9.4 μg/kg) in dried herbal infusions using the SPE method. SPE is a more expensive technique since it requires columns for each sample, making it difficult to apply to a large number of samples; also, extra solvents are used to condition the column and elute the analytes. In contrast, Gonçalves et al. [31] and Cirlini et al. [33] extracted TAs with solid–liquid extraction technique (SLE) obtaining lower LOQs, 1.2 μg/kg and 0.5 μg/kg, respectively. Despite the method’s simplicity and speed, dried herbal infusions are a complex matrix and, without a proper clean-up, could compromise the UHPLC system. For example, Dzuman et al. [32] applied SLE followed by d-SPE and had good sensitivity, with LOQs ranging between 1–5 μg/kg for 21 TAs. Thus, the developed methodology based on QuEChERS offered a greener, faster, and cheaper technique with comparable LOQs to other studies in the same matrix and included anisodamine and homatropine, TAs still not regulated in herbal infusions, with a rapid and easy technique based on QuEChERS.

##### Trueness and Precision

Table 3 shows the results of recovery, repeatability, and precision in validated spiked levels for the different tropane alkaloids in a blank dried herbal infusion sample spiked at eight different concentration levels. According to Directive 96/23/EC [40], when certified reference materials are unavailable, the recovery of additions of known amounts of the analytes to a blank matrix can be used to determine the trueness of the measurements. Concerning recovery, the method provides good recoveries for all four plant toxins within the appropriate range established by the Commission Regulation EC No. 401/2006 [42], ranging between 82 to 105%. For atropine, the recoveries ranged between 94 and 102%, while for scopolamine they ranged between 82 and 104%. Compared to other methods (Table 1), these recoveries are similar to those reported by León et al. [30] using the QuEChERS method (87–111%). These recoveries had a lower range than those reported by Romero-Torres et al. [34] (75–128%) and González-Gómez et al. [29] (56–104%) in dried herbal infusions using the SPE method.

Precision was evaluated in terms of repeatability and inter-day precision and was expressed as relative standard deviation (RSD). Regarding repeatability, it was evaluated by the relative standard deviation (RSD_r_) for all toxins, using the same sample, and the same operator within a short time. As it is shown in Table 3, good repeatability was obtained with all values lower than or equal to 11%, being suitable considering the criteria established by Commission Directive 96/23/EC. The highest RSD_r_ is for atropine at a spiked level of 5 μg/kg (10.99%) and scopolamine at a spiked level of 10 μg/kg (10.88%). These results are better than those reported by León et al. [30] (<13%), González-Gómez et al. [29] (<13%), and Romero-Torres et al. [34] (<25%).

Relative standard deviation (RSD_R_) was used to assess the method’s intra-day precision over three different analysis days, three distinct concentration levels, and different operators. The RSD_R_ values are acceptable, ranging between 3.1 and 13.1%. Homatropine was the TA with the higher values for RSD_R_, and consequently, with lower inter-day precision, while scopolamine had the highest inter-day precision. These results are better than those reported by González-Gómez et al. [29] (<16%) and Romero-Torres et al. [34] (<26%).

##### Specificity

By analyzing 20 blank samples (with the criterion of S/N < 30% of the blank sample spiked at LOD) and looking for interferences in the region of interest (retention time between 3.20 and 3.70 min) where the target analytes are expected to elute, the specificity of the approach was assessed. The results demonstrate that, for the four TAs under study, there was no signal or peak at the expected retention time of the analytes, demonstrating the absence of any matrix or chemical interferences. In parallel with the extractions, a reagent blank was performed, consisting in using all the reagents of the extraction, but without the sample. Results showed no interferences at the expected retention time of the analytes, indicating no effect of equipment or materials on the analytical response.

##### Matrix Effect

The matrix effect (ME) is caused by co-extracted compounds that negatively affect the analytical performance of target analytes. The method causes a signal suppression for atropine, anisodamine, and homatropine (Table 2). ME was soft for atropine (*SSE* = 89.2%) and medium for anisodamine (*SSE* = 62.6%) and homatropine (*SSE* = 64.6%). For scopolamine, the method causes a signal enhancement but is considered negligible (*SSE* = 105%). The developed technique generally causes a low matrix effect. The signal enhancement was found by Gonçalves et al. [31] and Romera-Torres et al. [34] for all TAs in tea and herbal infusions, with medium to strong effect, using SLE and SPE, respectively (Table 1). Certain spices were included in our study because they are also herbs. In spices, González-Gómez et al. [29] found a signal suppression, with a medium to a strong ME, ranging from 22 to 63% for scopolamine to 15 to 49% for atropine.

In summary, compared to the studies already published, the development of this methodology with UHPLC-ToF-MS provides excellent linearity, sensitivity, specificity, and trueness for TA determination in herbal infusions, including atropine, scopolamine, anisodamine, and homatropine.

### 2.2. Tropane Alkaloids in Commercial Samples of Herbal Infusions

To evaluate the presence of TAs in herbal infusions, 17 samples of different dried herbs available on the Portuguese markets were analyzed using the developed method. Each sample was extracted in duplicate.

**Figure 3:**
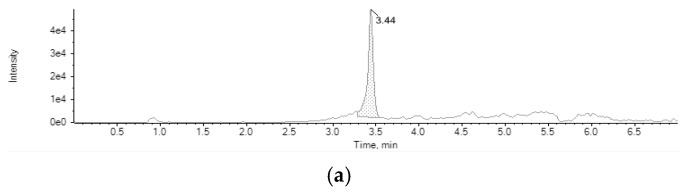

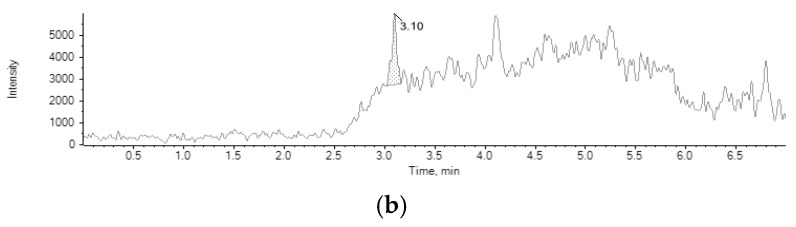
(**a**) Chromatogram of lemon balm *(Melissa officinalis* L.) sample contaminated with atropine (38.11 µg/kg), and (**b**) chromatogram of stinging nettle *(Urtica dioica* L.) sample contaminated with homatropine.

Atropine was found in one sample of *Melissa officinalis* L., commonly known as lemon balm, with a concentration of 38.11 µg/kg (Figure 3a). This level is higher than ML established for the sum of atropine and scopolamine (25 µg/kg) in herbal infusions [27], being a food safety and health concern. In Italy, a high level of atropine was also found by Cirlini et al. [33] in a mix of lemon balm, fennel, and malva with 25 µg/kg of atropine and 50 µg/kg of scopolamine, exceeding the ML established. Atropine was found in one sample of dandelion with turmeric above the maximum limit (69 µg/kg) [33]. Two further samples also had the same TA, but below the LOD (0.5 µg/kg) [33]. For instance, atropine and scopolamine were not identified in Spain when León et al. [30] assessed 11 samples, including rooibos, chamomile, red tea, black tea, green tea, white tea, linden, horsetail, and one infusion including a combination of herbs.

Homatropine was found in one sample of *Urtica dioica* L., known as stinging nettle, with a concentration of 10.98 µg/kg (Figure 3b). The presence of homatropine was not previously reported in herbal infusions. No limits are established for homatropine in any foodstuffs. Tropane alkaloids other than atropine and scopolamine were found by Romera-Torres et al. [34] in six samples of herbal infusions, where pseudotropine, physoperuvine, and tropine were the most detected. In addition, one of the samples corresponding to cocoa leaves presented a concentration of 27 μg/kg of atropine [34].

Even though the number of positive samples is limited (11%) and the data cannot accurately depict the distribution of TA in dried herbs used in Portugal for infusions, they do provide some insight into the problem. Since tea and herbal infusions have been widely consumed throughout the world and the evidence confirms that TA contamination may be present, the monitoring of these chemical contaminants is warranted.

The occurrence of TAs in the European market was performed by EFSA in 2016, where 121 samples of dry herbal tea were collected between June 2015 and August 2016 [43]. Seventy percent of the analyzed samples contained one or more TAs above the LOD (between 0.05 and 0.2 µg/kg), with atropine and scopolamine being the most often found TAs, with the highest total concentration of 428.5 µg/kg [43].

### 2.3. Antioxidant Capacity

Four experiments were carried out in this study to characterize the antioxidant properties of herbal infusions more accurately. The results are expressed in Table 4 regarding the DPPH radical scavenging capacity, β-carotene bleaching assay, TPC, and TFC.

Regarding the DPPH radical scavenging assay, lemon balm from brand B showed the highest inhibition percentage of DPPH radical, corresponding to 847.89 ± 5.18 µg TE/mL, with significant superiority (*p* < 0.05), followed by green tea (256.57 ± 7.68 µg TE/mL), Greek lemon verbena (670.50 ± 2.07 µg TE/mL), and yerba mate (642.63 ± 5.18 µg TE/mL). Moreover, peppermint from three different brands showed high values of antioxidant capacity through the DPPH radical scavenging assay.

Concerning β-carotene bleaching assay, peppermint form brand C presents the highest *AAC* (562.93 ± 32.47), with significant superiority (*p* < 0.05), followed by yerba mate (*AAC* = 522.11 ± 4.81). Nevertheless, the three samples of peppermint showed high *AAC* (between 408.45 ± 106.23 and 562.93 ± 32.47) as did the two samples of lemon balm (*AAC* = 322.38 ± 108.45 and 393.28 ± 7.82).

All the herbal infusions presented a high polyphenolic content, which is a good indicator of antioxidant capacity. Lemon balm from brand B presented the highest TPC (682.14 ± 0.79 µg GAE/mL), while yerba mate presented the highest TFC (1031.10 ± 7.78 µg EE/mL), though both samples of peppermint and lemon balm presented high TPC and TFC. 

Milk thistle infusion presented the lowest antioxidant capacity in the DPPH radical scavenging assay (27.73 ± 0.67 µg TE/mL) and the lowest content of phenolics and flavonoids, while ginger infusion presented the lowest antioxidant capacity through the β-carotene bleaching assay (*AAC* = 74.40 ± 69.15).

Peppermint infusions from three different brands were assessed, and results showed that the total polyphenolic and total flavonoid contents were significantly different (*p* < 0.05), although no significant differences (*p* > 0.05) were observed in DPPH radical scavenging activity or in β-carotene bleaching. These dissimilarities can be due to differences in the variability of species and different edaphoclimatic conditions. Regarding the two different brands of lemon balm, no significant differences (*p* > 0.05) were observed.

Even though DPPH and TPC are frequently used assays for determining the antioxidant activity of tea and herbal infusions, a review of the literature showed that the results are expressed in different units, different procedures have been used, and infusions have been prepared with different methods; thus, we were unable to compare those results with those from the current study [44,45]. For example, Rząsa-Duran et al. (2022) [46] assessed the antioxidant capacity of yerba mate from different origins, reporting an inhibition of the DPPH radical ranging from 20.20 to 61.29%. Regarding the polyphenolic content, the authors used different procedures to determine TPC and TFC, with results varying between 22.05 and 86.23 mg GAE/mL for TPC and between 36 and 475 mg quercetin equivalent/mL for TFC. For instance, Boneza et al. (2018) [47] noted a total phenolic content ranging from 5.05 to 26.87 mg GAE/g DW for the four lemon balm cultivars within their study but used a different procedure. 

According to what we observe, the β-carotene bleaching assay is not a frequently used assay to evaluate the antioxidant capacity of herbal infusions. However, Robalo et al. (2022) [48] performed the assay in green tea from four different brands. The results indicated that green tea has a high antioxidant capacity since the *AAC* ranged between 600 to 746 [48], higher than our results (*AAC* = 183.10 ± 66.40). Comparing the results of the DPPH radical assay, our result for green tea infusion showed a higher antioxidant capacity; however, similar total phenolics and flavonoids content was found.

In summary, all the herbal infusions presented a good source of antioxidants. Nevertheless, from the studied herbal infusions, yerba mate (*Ilex paraguariensis* L.), lemon balm (*Melissa officinalis* L.), and peppermint (*Mentha x piperita* L.) were revealed to have the greatest antioxidant capacity through both DPPH radical scavenging and β-carotene bleaching assays, and they were richer in phenolic compounds. Previously, Gorjanovic et al. (2012) [49] assessed the antioxidant capacity of different tea and herbal infusions, concluding that green tea, peppermint, and lemon balm had the highest antioxidant capacity through the DPPH radical assay and the highest total phenolics content. More recently, Bakrim et al. (2022) [50] have compared the antioxidant capacity of different plants of *Menth* genus, and peppermint showed the best ability to scavenge the DPPH radical.

## 3. Conclusions

This study provides two different perspectives on herbal infusions: their possible contamination by plant toxins and their antioxidant capacity due to the presence of antioxidant compounds.

On one hand, this study evaluated the presence of tropane alkaloids in herbal infusions. For that, an analytical screening quantitative method based on QuEChERS followed by ultra-high-performance liquid chromatography coupled with high-resolution time-of-flight mass spectrometry was developed and validated for the simultaneous detection of four tropane alkaloids in herbal infusions, including atropine and scopolamine.

The validated method provided good analytical performance, with good recovery (82 to 105%), repeatability (RSD_r_ < 11%), and precision (RSD_R_ < 13%) in agreement with criteria established by Commission Recommendation EU No. 2015/976 for the monitoring of the presence of tropane alkaloids in food [51]. The LOQ for atropine and scopolamine was 5 µg/kg, which is lower than the maximum levels regulated by the EU (25 µg/kg for the sum of atropine and scopolamine). In addition, the QuEChERS method allows for an efficient TA extraction and the removal of substances that may interfere with the HPLC system, being quick, easy, and simple to apply to a large number of samples in a short amount of time and an alternative to SPE methods.

When the method was applied to seventeen herbal infusions, atropine was detected in one sample of lemon balm, with a concentration higher than the maximum level regulated in the EU. In addition, homatropine was found in stinging nettle; however, this compound does not have stablished maximum permitted levels in the EU. 

On the other hand, the antioxidant capacity of herbal infusions was assessed. From the studied herbal infusions, yerba mate (*Ilex paraguariensis* L.), lemon balm (*Melissa officinalis* L.), and peppermint (*Mentha x piperita* L.) were revealed to have the greatest antioxidant capacity through DPPH radical scavenging and β-carotene bleaching assays, and they were richer in phenolic compounds.

Comparing both results and striking a balance between the antioxidant capacity and the presence of tropane alkaloids, yerba mate and peppermint are promising sources of antioxidants, with the potential to be applied in food and active food packaging as an alternative to synthetic antioxidants and in the aim toward a sustainable and circular economy approach. Though green tea has been the most studied plant for its antioxidant properties, other herbal infusions have great potential to be applied in the food industry. However, the presence of other contaminants should be addressed, such as mycotoxins and pesticide residues in order to guarantee food safety.

## 4. Materials and Methods

### 4.1. Samples and Sampling Procedure

Seventeen commercial samples of dried herbal plants for infusions and tea were randomly purchased in different supermarkets in Portugal between April and May of 2022, with different origins and from organic and conventional agriculture (Table S1). To ensure homogeneity, the samples were ground (Micro Fine Mill Grinder Culatti MFC with a filter of 1.5 mm) and thoroughly combined. The samples were stored in the dark at room temperature and in a dry place until further analysis.

### 4.2. Determination of Tropane Alkaloids

#### 4.2.1. Chemicals and Reagents

Atropine ((±)-hyoscyamine), (±)-scopolamine hydrochloride, anisodamine, and homatropine hydrobromide were bought from Sigma-Aldrich as standards for tropane alkaloids (Madrid, Spain). For internal standards (IS), atropine-d5 and scopolamine-13C, d3 Hydrobromide were purchased from Toronto Research Chemicals Inc. (Toronto, ON, Canada). Before use, all standard solutions were maintained at room temperature for 30 min before being stored in amber vials in the dark at −20 °C.

Methanol (MeOH), acetonitrile (ACN), both HPLC-gradient grade, and formic acid were purchased from Merck (Darmstadt, Germany). Milli-Q plus system from Millipore (Molsheim, France) was used to purify the water, and it had a resistivity of 18.2 M cm.

For QuEChERS, magnesium sulfate and trisodium citrate dihydrate were purchased from PanReac (Barcelona, Spain), sodium chloride from Fluka (Seelze, Germany), and sodium citrate dibasic sesquihydrate from Sigma-Aldrich (Madrid, Spain). Agilent Technologies (Santa Clara, CA, USA) supplied the primary secondary amine-bonded silica (PSA) that was used for clean-up, in combination with anhydrous magnesium sulfate acquired from PanReac (Barcelona, Spain).

#### 4.2.2. Preparation of Standard Solutions

Four tropane alkaloids’ stock standard solutions were prepared for each one by diluting them in methanol [52]. The stock solution of homatropine hydrobromide was prepared at 10 mg/mL, while scopolamine hydrochloride was prepared at 10 µg/mL. Atropine and anisodamine solutions were prepared at a concentration of 1 mg/mL. The working solution for calibration was therefore prepared using these four stock solutions.

A calibration work solution was prepared in methanol with a mixture of atropine, scopolamine, anisodamine, and homatropine stock standard solutions to achieve a final concentration of 100 ng/mL for each analyte.

For internal standards, a stock solution of each IS was prepared in methanol at a concentration of 100 µg/mL. Then, using methanol as the solvent, a mixture of two IS was prepared at a final concentration of 1 µg/mL.

#### 4.2.3. Extraction Procedure

**Figure 4:**
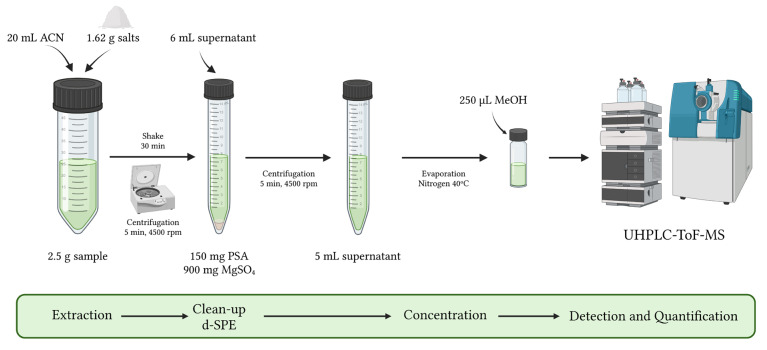
Summarized scheme of the analytical method for determination of TAs in herbal infusions.

Tropane alkaloids were extracted using a modified version of the QuEChERS method (Figure 4). Approximately 2.5 g of sample (2.5 ± 0.1 g) was weighted in 50 mL Falcon tubes. Then, 200 μL of internal standards solution (1 μg/mL) was added, followed by 20 mL of acetonitrile. The samples were mixed for 1 min in vortex and then shaken for 30 min using a Kotterman 4010 Orbital Shaker (Uetze/Hanigsen, Germany). The extraction salt mixture for the liquid–liquid partitioning stage was then added (1.62 g) and vortexed for 1 min. One gram of magnesium sulfate, 0.25 g of sodium chloride, 0.25 g of trisodium citrate dihydrate, and 0.12 g of disodium hydrogen citrate sesquihydrate were all included in this mixture. The samples were then centrifuged for 5 min at 5 °C at 3622× *g.*

In the dispersive solid-phase extraction (d-SPE) step, 6 mL of the extract was transferred into a tube containing 150 mg primary secondary amine (PSA) sorbent and 900 mg anhydrous MgSO_4_, followed by centrifugation at 2580× *g* for 2 min at 5 °C.

Five mL of extract was then transferred into a 15 mL Falcon tube and evaporated at 40 °C, over a stream of nitrogen. The residues were redissolved with 250 μL of pure MeOH, vortexed for 30 s, followed by 10 min in an ultrasonic bath. The redissolved extract was lastly filtered through a 0.2 μm PVDF mini-uniprep^TM^ before being injected into the UHPLC-ToF-MS system.

#### 4.2.4. UHPLC–ToF-MS Parameters

The detection and quantification of TA were performed using a Nexera X2 Shimadzu UHPLC linked to a 5600+ ToF-MS detector (SCIEX, Foster City, CA, USA) equipped with a Turbo Ion Spray electrospray ionization source operating in positive mode (ESI+).

A column Acquity UPLC BEH C18 (2.1 mm × 100 mm, 1.7 μm) was used for chromatographic separation and maintained at 20 °C. The autosampler was kept at 10 °C to refrigerate the samples. A volume of 20 μL of extract solution was injected into the column at a flow rate of 0.3 mL/min. The mobile phase consisted of water [A] and acetonitrile [B], both acidified with 0.1% formic acid, with the following gradient program: start with 5% [B]; 0.01–6 min from 5% to 80% [B]; 6–6.5 min from 80% to 3% [B]; and kept until the end of the run with 3% [B], with total run time of 10 min. 

Using Analyst^®^ TF software (SCIEX, Foster City, CA, USA) and the following parameters for mass spectrometry, the acquisition was carried out in a full scan from 100 to 750 Da: ion source voltage of 5500 V; source temperature of 575 °C; curtain gas (CUR) of 30 psi; Gas 1 and Gas 2 of 55 psi; and declustering potential (DP) of 100 V. In order to provide precise mass resolution, the ToF-MS detector was calibrated every seven injections in the method’s mass range. 

#### 4.2.5. Identification of Tropane Alkaloids

PeakView^TM^ and MultiQuant^TM^ software were used for the identification and data processing of tropane alkaloids (SCIEX, Foster City, CA, USA). The PeakView^TM^ program presents the isotope match automatically. Three factors and their respective equations (Equations (1)–(3)) were utilized to determine whether the identification criteria of the analytes were accepted: (1) maximum relative retention time deviation (ΔRRT) of 2.5% (Equation (1)); (2) difference in the isotope pattern with a tolerance of 10% (Equation (2)); and (3) exact mass deviation (Δm) with a tolerance of 5 ppm (Equation (3)).

(1) $$∆RRT=\frac{RT_{tropane\;alkaloid}}{RT_{internal\;standard}},$$

where *RT_internal standard_* is the retention time of the appropriate internal standard, depending on the analyte, and *RT_tropane alkaloid_* is the retention time of each analyte.

(2) $$RTT=\left(\frac{RTT_{spiked\;sample}-RTT_{standard}}{RTT_{standard}}\right)\times 100$$

(3) $$∆m\;\left(ppm\right)=\left(\frac{exact\;mass-detected\;mass}{exact\;mass}\right)\times 10^{6}$$

#### 4.2.6. Validation of the UHPLC-ToF-MS Method

The parameters used for validation of the methodology were concentration range, linearity, limit of detection (LOD), limit of quantification (LOQ), recovery assays at different spiking levels, precision (repeatability and precision inter-day), and matrix effect (ME). The method was validated for the determination of four tropane alkaloids: atropine, scopolamine, anisodamine, and homatropine. Blank samples of green tea (not contaminated with any of the evaluated TAs) was used for full validation of the method.

##### Spiking Experiments

In order to obtain a concentration range between 1.25 and 50 μg/kg of the target analytes, a blank sample of green tea (2.5 g) was spiked with eight different levels using 31.2 µL to 1250 µL of calibration of the work solution (Section 2.1) to construct a matrix-matched calibration curve. The extraction procedure was then carried out as explained in Section 2.3. These concentration levels include the maximum level imposed for the sum of atropine and scopolamine for herbal infusions (dried product) for human consumption (25 μg/kg) established in EC Regulation No. 1881/2006, recently updated by the EC Regulation No. 2021/1408 [27].

The ratio between the analyte area and the equivalent IS area and the concentration (µg/kg) were used to construct the curves by linear regression analysis. The concentrations that result in signal-to-noise ratios (S/N) of less than 3 and 10, respectively, were identified as LOD and LOQ. Recovery was determined by repeated analyses (*n* = 6) of a spiked blank green tea, at different levels. Blank samples of herbal infusions were spiked at various levels, taking into consideration the ML of tropane alkaloids, to determine repeatability (RSD_r_) and precision inter-day (RSD_R_). Extraction in the case of RSD_r_ was performed by two independent operators on three different days. Additionally, twenty blank samples of blank green tea were also analyzed in order to evaluate the specificity of the procedure.

##### Matrix Effect

The matrix effect (ME) was calculated using the signal suppression–enhancement (*SSE*) (Equation (4)) by comparing the slopes of the calibration standard solution and the matrix-matched calibration curve with the spiked green tea sample. For that, a calibration curve in the range of 1.25–30 μg/kg (six points, two injections each) for the four considered tropane alkaloids was prepared in methanol.

(4) $$SSE\;\left(\%\right)=\frac{Slope_{\mathrm{matrix}-\mathrm{matched}\;\mathrm{calibration}\;\mathrm{curve}}}{Slope_{standard\;calibration\;curve}}\times 100,$$

*SSE* > 100% and *SSE* 100% were taken into consideration for signal enhancement and suppression, respectively. The matrix effect could be also classified as negligible ([0%]–[±10%]), soft ([±10%]–[±20%]), medium ([±20%]–[±50%]), and strong ([±50%]), according to Matuszewski et al. [51].

### 4.3. Determination of Antioxidant Capacity

The antioxidant capacity of herbal infusions was evaluated and compared using the DPPH radical scavenging activity assay and the and β-carotene bleaching assay. Moreover, the total phenolics and total flavonoids were determined. In addition, the total phenolic content and total flavonoid content were determined. These four assays were performed using a UV-Vis Spectrophotometer (U-2810, Hitachi, Digilab, Tokyo, Japan) to measure the samples’ absorbance against water.

#### 4.3.1. Preparation of Herbal Infusions

Briefly, 2 g of each dried herb for infusion were weighed, and 200 mL of water at 75 °C was added and soaked for 3 min. Then, the infusions were filtered with a syringe (Acrodisc^®^ Premium, GHP 0.2 μm) and protected from light, until further analysis.

#### 4.3.2. DPPH Radical Scavenging Assay

The DPPH radical (2,2-diphenyl-1-picryl-hydrazyl) assay was applied following the method described by Moure et al. [53] and modified by Andrade et al. [54].

A 14.2 g/mL methanolic solution of DPPH (Sigma-Aldrich) was prepared. In brief, 2 mL of a DPPH solution was mixed with 50 μL of the sample. After homogenization, the solutions were protected from light for 30 min. Absorbance was measured at 515 nm. The inhibition percentage (IP%) of DPPH was calculated by Equation (5).

(5) $$IP\;\left(\%\right)=\frac{Ac-As}{Ac}\times 100$$

where *Ac* represents the absorbance of the control (water) and *As* represents the absorbance of the sample. 

A calibration curve ($y=0.3086x+0.1894,\;r^{2}=0.9983$) was drawn up using different concentrations of Trolox (6-hydroxy-2,5,7,8-tetramethylchroman-2-carboxylic acid) (Sigma-Aldrich), with a working range of 5–250 µg/mL. Results were expressed as µg Trolox equivalent (TE)/mL of infusion.

#### 4.3.3. β-Carotene Bleaching Assay

The β-carotene bleaching assay was carried out in accordance with Miller’s specifications [55] and improved by Andrade et al. [54]. First, a solution of 2 mg/mL of β-carotene in chloroform (Sigma-Aldrich) was prepared. Then, 1 mL of the β-carotene solution was mixed with 20 mg of linoleic acid (Sigma-Aldrich) and 200 mg of Tween^®^ 40 (Sigma-Aldrich) to prepare an emulsion. The chloroform was evaporated at 40 °C in a rotatory evaporator (Büchi Rotavapor R-114). Then, 50 mL of ultra-pure water was added, and the solution was vigorously shaken. Lastly, 5 mL of the β-carotene emulsion was added to 200 µL of each sample. The samples were kept in a heating bath (Büchi B-491) at 50 °C for 120 min. Afterwards, the absorbance of the samples was measured at 470 nm. For control samples, 200 µL of the solvent used in the extraction process was used, and the absorbance was measured before and after the 120 min. The antioxidant activity coefficient (*AAC*) was calculated by Equation (6).

(6) $$AAC=\frac{As_{120}-Ac_{120}}{Ac_{0}-Ac_{120}}\times 1000$$

where *As*_120_ is the sample’s absorbance at 120 min, *Ac*_120_ is the control’s absorbance at 120 min, and *Ac*_0_ is the control’s absorbance at 0 min.

#### 4.3.4. Total Phenolic Content (TPC)

According to Erkan et al. (2008) [56,57], the Folin–Ciocâlteu reagent assay, which is frequently used to evaluate total phenolic content (TPC), was performed. Briefly, 1 mL of each sample was mixed with 7.5 mL of Folin–Ciocâlteu reagent (10%, *v*/*v*) (Sigma-Aldrich). After 5 min, 7.5 mL of sodium carbonate aqueous solution (60 mg/mL) (Sigma-Aldrich) was added. The solutions were homogenized and then left at room temperature for 120 min in the dark. Finally, absorbance was measured at 725 nm.

Furthermore, a calibration curve ($y=0.0059x-0.1391,\;r^{2}=0.9933$) using gallic acid (Sigma-Aldrich) as a standard was drawn with a working range of 25–150 µg/mL. Results were expressed in mg gallic acid equivalent (GAE)/mL of infusion.

#### 4.3.5. Total Flavonoid Content (TFC)

The method outlined by Yoo et al. (2008) [58] was used to determine the TFC. In brief, 1 mL of sample was combined with 4 mL of ultra-pure water. The mixture was then homogenized with 300 µL of an aqueous solution of sodium nitrite (5%, *w*/*v*) (Supelco). After waiting for five minutes, 600 µL of an aqueous aluminum chloride solution (10%, *w*/*v*) (Sigma-Aldrich) was added, and the mixture was once more homogenized. After 6 min, 2 mL of an aqueous solution of sodium hydroxide (1 M) (Sigma-Aldrich) was added. The samples were then homogenized, and the absorbance at 510 nm was measured. A reagent blank was prepared using distilled water. The entire process took place at room temperature.

A calibration curve ($y=0.0018x+0.0181,\;r^{2}=0.9954$) was built for the TFC assay by plotting different concentrations of epicatechin (Sigma-Aldrich) (5–200 µg/mL). Results were expressed in mg epicatechin equivalent (EE)/mL of the sample.

#### 4.3.6. Statistical Analysis

Statistical analysis of the data was performed through the Kruskal-Wallis H test, using the software IBM^®^ SPSS^®^ Statistics, version 28.0.1.1. The Kruskal-Wallis H test is the non-parametric alternative to the One Way ANOVA when the normality of data or homogeneity of variances was not validated. The Kruskal-Wallis H test was applied to the comparison between the different infusions, in the four trials carried out. Significance was defined at *p* < 0.05. Results concerning the statistical evaluation of the antioxidant capacity of the infusions were expressed as mean ± standard deviations (SD) of three replicates.

## Figures and Tables

**Table 1:** Compilation of methods to determine tropane alkaloids in herbal infusions from 2018 to 2023.

Plant Toxins	Food Matrix	Extraction Procedure	Chromatographic System	LOQ (μg/kg)	Ref.
Atropine, scopolamine	Spices	SPE	HPLC-QqQ MS/MS	1.9–9.4	[29]
TAs (atropine and scopolamine) and PAs (28 *)	Teas and herbs for infusions	QuEChERS	UHPLC-Q-Orbitrap MS/MS	5	[30]
Atropine, scopolamine	Cereals, tea, and herbal infusions	SLE	HPLC-MS/MS	1.2	[31]
TAs (21 *) and PAs (33 *)	Mixed herbal tea, sorghum, and oregano	SLE+ d-SPE	UHPLC-QTRAP MS/MS	1–5	[32]
TAs (anisodamine, atropine, homatropine, scopolamine)	Herbal teas and infusions	SLE	UHPLC-QqQ-MS/MS	0.5	[33]
TAs (13 compounds, including anisodamine, atropine, homatropine, and scopolamine)	Teas and herbal teas	SPE	UHPLC-Orbitrap MS/MS	5–15	[34]

* The numbers between brackets correspond to the number of compounds analyzed in each study; d-SPE—Dispersive-Solid Phase Extraction; HPLC—High-Performance Liquid Chromatography; HRMS—High-Resolution Mass Spectrometry; LOQ—Limit of Quantification; MS/MS—tandem Mass Spectrometry; PAs—Pyrrolizidine Alkaloids; QuEChERS—Quick, Easy, Cheap, Effective, Rugged and Safe; SLE—Solid-Liquid Extraction; SPE—Solid-Phase Extraction; TAs—Tropane Alkaloids; UHPLC—Ultra-Hight Performance Liquid Chromatography.

**Table 2:** Linearity, sensitivity, and matrix effect of the UHPLC-ToF-MS method for the simultaneous determination of tropane alkaloids in herbal infusions.

Toxin	Internal Standard	Linear Range	Calibration Curve Parameters			LOD	LOQ	*SSE*
		(µg/kg)	r^2^	Slope	Interception	(µg/kg)	(µg/kg)	(%)
Atropine	Atropine-d5	5.0–50	0.9958	0.0104	0.0217	2.5	5	89.2
Scopolamine	Scopolamine 13C,d3	5.0–50	0.9976	0.0152	0.0176	2.5	5	105
Anisodamine	Scopolamine 13C,d3	15.0–50	0.9867	0.0139	−0.1108	10	15	62.6
Homatropine	Atropine-d5	5.0–50	0.9930	0.0041	0.0188	2.5	5	64.6

r^2^—determination coefficients; LOD—limit of detection; LOQ—limit of quantification; *SSE*—signal suppression-enhancement.

**Table 3:** Outcomes of validation for four plant toxins, using green tea as a blank matrix, including recovery (Rec), relative standard deviation repeatability (RSD_r_), and relative standard deviation of precision inter-day (RSD_R_) at various spiking levels.

Toxin	Ion	Retention Time (min)	Spiked Level (µg/kg)	Rec (%)	RSD_r_ (%)	RSD_R_ (%)
Atropine	[M + H]^+^	3.64	5	99.07	10.99	11.28
			10	94.44	5.10	7.92
			20	99.52	7.30	
			30	102.30	2.75	11.07
			40	100.19	8.07	
			50	98.85	0.92	
Scopolamine	[M + H]^+^	3.33	5	82.04	9.27	9.37
			10	104.21	10.88	8.77
			20	103.58	3.95	
			30	102.47	2.95	3.12
			40	96.88	2.11	
			50	100.36	1.45	
Anisodamine	[M + H]^+^	3.26	15	102.06	4.57	11.38
			20	104.93	5.25	
			30	90.75	3.96	5.21
			40	103.10	3.89	
			50	97.77	3.10	
Homatropine	[M + H]^+^	3.31	5	95.83	8.93	13.10
			10	103.65	5.95	11.45
			20	104.17	9.59	
			30	97.48	2.51	11.25
			40	100.70	5.66	
			50	103.90	2.96	

**Table 4:** Antioxidant capacity of different herbal infusions.

Herbal Infusions	DPPH (µg TE/mL)	β-Carotene (*AAC*)	TPC (µg GAE/mL)	TFC (µg EE/mL)
Anise	109.58 ± 1.12 ^f^	156.46 ± 30.06 ^e^	43.76 ± 0.10 ^l^	47.66 ± 2.99 ^i^
Chamomile	117.23 ± 6.35 ^f^	203.44 ± 77.46 ^e^	114.32 ± 0.10 ^j^	62.47 ± 1.60 ^i^
Fennel	119.84 ± 1.12 ^f^	275.51 ± 54.12 ^d^	78.16 ± 0.20 ^k^	54.71 ± 2.19 ^i^
Ginger	137.59 ± 0.56 ^e^	74.40 ± 69.15 ^f^	113.41 ± 0.30 ^j^	99.00 ± 1.40 ^h^
Greek lemon verbena	670.50 ± 2.07 ^b^	275.51 ± 54.12 ^d^	317.65 ± 1.18 ^h^	455.03 ± 2.59 ^e^
Indian senna	94.09 ± 3.67 ^g^	291.08 ± 64.18 ^d^	178.34 ± 0.17 ^j^	144.28 ± 0.80 ^h^
Lemon balm A	264.13 ± 1.00 ^d^	322.38 ± 108.45 ^d^	497.62 ± 0.39 ^c^	705.68 ± 4.79 ^d^
Lemon balm B	847.89 ± 5.18 ^a^	393.28 ± 7.82 ^c^	682.14 ± 0.79 ^a^	938.57 ± 10.57 ^b^
Yerba mate	642.63 ± 5.18 ^b^	522.11 ± 4.81 ^b^	569.38 ± 0.39 ^b^	1031.10 ± 7.78 ^a^
Milk thistle	27.73 ± 0.67 ^h^	184.66 ± 73.03 ^e^	32.54 ± 0.10 ^l^	9.15 ± 0.40 ^j^
Narrow-leaved purple coneflower	249.01 ± 1.00 ^d^	305.16 ± 106.23 ^d^	276.72 ± 0.59 ^i^	424.42 ± 3.59 ^f^
Peppermint A	252.08 ± 0.67 ^d^	427.23 ± 132.79 ^c^	392.44 ± 0.71 ^d^	446.42 ± 2.79 ^e^
Peppermint B	248.54 ± 1.67 ^d^	408.45 ± 106.23 ^c^	323.14 ± 3.56 ^g^	798.78 ± 11.97 ^c^
Peppermint C	397.05 ± 0.00 ^c^	562.93 ± 32.47 ^a^	356.15 ± 1.04 ^f^	550.38 ± 4.59 ^d^
Stinging nettle	174.15 ± 9.35 ^h^	247.26 ± 121.72 ^d^	121.61 ± 0.17 ^j^	132.43 ± 2.39 ^h^
Green tea	256.57 ± 7.68 ^d^	183.10 ± 66.40 ^e^	365.10 ± 0.71 ^e^	158.67 ± 8.38 ^g^
Thyme	149.03 ± 1.12 ^e^	188.78 ± 64.94 ^e^	75.48 ± 0.00 ^k^	86.03 ± 2.19 ^h^

The results are expressed as mean ± SD, per mL of infusion. Different letters within the same row indicate statistical differences (*p* < 0.05).

## Data Availability

Data sharing is not applicable to this article.

## Supplementary Materials

The following supporting information can be downloaded at: https://www.mdpi.com/article/10.3390/toxins15040245/s1, Table S1: Information about samples of dried plants for infusion and their characteristics.
