# Supplementary material for: The Bright and Dark Sides of Herbal Infusions: Assessment of Antioxidant Capacity and Determination of Tropane Alkaloids
Source: Toxins (Basel). 2023 Mar 27;15(4):245. doi: 10.3390/toxins15040245 (PMC10144634; doi:10.3390/toxins15040245)
Supplement: Supplementary file 1 [file toxins-15-00245-s001.zip › toxins-2219599-SI.pdf]

Supplementary Materials: The Bright and Dark Sides of Herbal Infusions: Assessment of Antioxidant Capacity and Determination of Tropane Alkaloids

Ana Rita Soares Mateus, Carmen Crisafulli, Matilde Vilhena, Sílvia Cruz Barros, Angelina Pena and Ana Sanches Silva

Table S1. Information about samples of dried plants for infusion and their characteristics.

Sample	Common name	Scientific name	Origin	Biological agriculture	Expiration date
A	Peppermint	*Mentha x piperita* L.	EU	No	feb/25
B	Milk thistle	*Silybum marianum* L.	Portugal	No	march/25
C	Lemon balm	*Melissa officinalis* L.	EU	No	march/25
D	Chamomile	*Matricaria Chamomilla* L.	Croatia	No	jan/25
E	Narrow-leaved purple cone-flower	*Echinacea angustifolia* DC.	EU	Yes	jan/24
F	Stinging nettle	*Urtica dioica* L.	EU	Yes	jan/24
G	Peppermint	*Mentha × piperita* L.	Austria	Yes	april/25
H	Indian Senna	*Cassia angustifolia* L.	India	No	march/25
I	Tea plant	*Camellia sinensis* L.	Not EU	No	april/25
J	Lemon Balm	*Melissa officinalis* L.	EU	Yes	nov/22
K	Yerba mate	*Ilex paraguariensis* A. St. Hil	EU	Yes	nov/22
L	Greek Lemon Verbena	*Aloysia citrodora* P.	EU	Yes	nov/22
M	Ginger	*Zingiber officinale* R.	EU	Yes	nov/22
N	Peppermint	*Mentha x piperita* L.	EU	Yes	nov/22
O	Fennel	*Foeniculum vulgare* Mill.	EU	Yes	nov/22
P	Thyme	*Thymus vulgaris* L.	EU	Yes	nov/22
Q	Anise	*Pimpinella anisum* L.	EU	Yes	nov/22
